# Awareness, support, and opinions of healthy food and drink policies: a survey of staff and visitors in New Zealand healthcare organisations

**DOI:** 10.1186/s12889-024-19693-2

**Published:** 2024-08-12

**Authors:** Sarah Gerritsen, Magda Rosin, Lisa Te Morenga, Yannan Jiang, Bruce Kidd, Stephanie Shen, Elaine Umali, Sally Mackay, Cliona Ni Mhurchu

**Affiliations:** 1 School of Population Health, University of Auckland, Private Bag 92019, Auckland Mail Centre 1142, Auckland, New Zealand; 2 Research Centre for Hauora and Health, Massey University, Wellington, New Zealand; 3 National Institute for Health Innovation, University of Auckland, Auckland, New Zealand; 4 Department of Public Health, University of Otago, Wellington, New Zealand; 5 Te Whatu Ora – Te Toka Tumai Auckland, Auckland, New Zealand; 6 George Institute for Global Health, Sydney, Australia

**Keywords:** Food policy, Evaluation, Workplace health, Healthy food availability, Food services, Equity, Survey, Healthcare, Hospital

## Abstract

**Background:**

In 2016, a voluntary National Healthy Food and Drink Policy (hereafter, “the Policy”) was released to encourage public hospitals in New Zealand to provide food and drink options in line with national dietary guidelines. Five years later, eight (of 20) organisations had adopted it, with several preferring to retain or update their own institutional-level version. This study assessed staff and visitors’ awareness and support for and against the Policy, and collected feedback on perceived food environment changes since implementation of the Policy.

**Methods:**

Cross-sectional electronic and paper-based survey conducted from June 2021 to August 2022. Descriptive statistics were used to present quantitative findings. Free-text responses were analysed following a general inductive approach. Qualitative and quantitative findings were compared by level of implementation of the Policy, and by ethnicity and financial security of participants.

**Results:**

Data were collected from 2,526 staff and 261 visitors in 19 healthcare organisations. 80% of staff and 56% of visitors were aware of the Policy. Both staff and visitors generally supported the Policy, irrespective of whether they were aware of it or not, with most agreeing that “Hospitals should be good role models.” Among staff who opposed the Policy, the most common reason for doing so was freedom of choice. The Policy had a greater impact, positive and negative, on Māori and Pacific staff, due to more frequent purchasing onsite. Most staff noticed differences in the food and drinks available since Policy implementation. There was positive feedback about the variety of options available in some hospitals, but overall 40% of free text comments mentioned limited choice. 74% of staff reported that food and drinks were more expensive. Low-income staff/visitors and shift workers were particularly impacted by reduced choice and higher prices for healthy options.

**Conclusions:**

The Policy led to notable changes in the healthiness of foods and drinks available in NZ hospitals but this was accompanied by a perception of reduced value and choice. While generally well supported, the findings indicate opportunities to improve implementation of food and drink policies (e.g. providing more healthy food choices, better engagement with staff, and keeping prices of healthy options low) and confirm that the Policy could be expanded to other public workplaces.

## Background

Malnutrition and adult obesity continue to increase globally, with stark health inequities in nutrition-related chronic conditions among those who are the most disadvantaged in societies [1]. Adult eating behaviours can be influenced by workplace food environment interventions [2–5], and so the past two decades have seen an increase in healthy food and drink policies in public sector workplaces. The World Health Organization states that governments have a “unique opportunity and responsibility to lead by example through the implementation of healthy public food procurement and service policies, requiring that all foods and beverages served or sold in public settings contribute to the promotion of healthy diets” (6, p. v). Indeed, many health organisations in high-income countries have implemented healthy food and drink policies as ‘role models’ or leaders for the public sector [7, 8] following high-profile criticism for limited food choices and permitting fast-food franchises to operate inside hospitals [9–11].

Healthy food and drink policies aim to improve the nutritional offerings onsite in workplace cafeterias, vending machine, and retail outlets. A randomised controlled trial in workplace cafeterias found that increasing the proportion of healthier options was a promising intervention to reduce energy purchased [12], and pricing and availability strategies are effective at improving the nutritional quality foods and beverages purchased from vending machines [13] Even though research shows that voluntary policy initiatives are less effective than regulatory approaches [14–16], Australian hospital healthy food and drink policies to date have been a mix of mandatory (ACT, WA, SA, NT states) and voluntary (NSW, QLD, VIC, TAS states), with most using a traffic light system to differentiate healthy from unhealthy products [17].

In New Zealand, a voluntary National Healthy Food and Drink Policy (hereafter referred to as “the Policy”) was released in 2016 and updated in 2019 [18] to encourage hospitals to demonstrate commitment to the health and wellbeing of visitors and staff by providing food and drink options for staff and visitors in line with national food-based dietary guidelines [19] and discouraging consumption of unhealthy options. The Policy uses a colour-coded categorisation system to classify products as green, amber, or red. Green foods and drinks “reflect a variety of foods from the four core food groups [fruit and vegetables, grain foods, milk and milk products, protein foods]”, “are low in saturated fat, added sugar and added salt, and are mostly whole and less processed” [18]. Amber foods and drinks, such as salted nuts and fruit juices, are “not considered part of an everyday diet but may have some nutritive value” and their portion is often restricted in the National Policy. Red foods and drinks are “of poor nutritional value and are high in saturated fat, added sugar, and/or added salt” and include items such as confectionery, deep-fried foods and sugar-sweetened beverages, and those that exceed portion size limits [18]. The traffic light categories are used to guide food providers when implementing the Policy, but are not used as interpretative front-of-package labels to guide customers towards healthier choices. The Policy states that the healthy green item foods and drinks “should make up at least 55% of food and drinks available for consumption”, less healthy amber items should “make up less than 45% of choices available”, and the unhealthy “red items are not permitted” [18]. Most packaged foods need to meet set nutrient criteria standards (e.g., a Health Star Rating (HSR) of at least 3.5 out of possible 5 stars), and portion size limits apply to some categories. Five years following the release of the Policy, eight (of 20) District Health Boards and the Ministry of Health had adopted it, with several preferring to retain or update their own institutional-level version [20].

A recent systematic review of healthy cafeteria initiatives in hospitals has called for more consumer behaviour research to improve implementation of such policies [21]. Thus the aim of this research was to evaluate staff and visitor satisfaction with the available food and drink options and their support or opposition to the Policy in all New Zealand hospital settings, and in other national health organisations, examining differences according to adoption of the Policy.

## Methods

### Design, settings and aims

The design of the study was a cross-sectional survey of staff and visitors in all of the 20 publicly funded regional district health boards (DHB) responsible for healthcare delivery in hospitals and clinical centres in their jurisdictions; and at national health organisations that had adopted the national policy. In total, 18 out of 20 DHBs and the Ministry of Health participated in the project (Waitematā DHB did not consent to survey distribution, although they did participate in the wider evaluation audit of food and drink products, and Waikato DHB declined to participate in all parts of the evaluation). The study is part of an independently funded evaluation of the adoption (discussed below), implementation (results outlined in the discussion), and impact of the Policy.

Predominantely quantitative data were collected in the survey, although free text ‘other’ response options were allowed for most questions, and a final open ended questions was included for participants to add “any further information” on the topic. The quantitative component provided general patterns and allowed for statistical comparisons, whereas the qualitative phase revealed opinions and reasons behind the patterns, strengthening the conclusions of the study.

We aimed to collect feedback from staff and visitors in public healthcare organisations about current food environments, and any perceived changes noticed by staff resulting from implementation of the Policy. To assess the equity implications of the Policy, we examined differences in staff and visitor responses by ethnicity and socioeconomic position. Given varying levels of adoption of the voluntary Policy, we also compared whether the Policy in each organisation (if they had adopted the national policy, had their own strongly-worded organisational policy, or their own weakly-worded organisational policy) affected the availability of healthy foods. The stratification variable for this comparison was obtained from a previous study where organisational policy comprehensiveness was determined through a quantitative content analysis of the written policies [20]. The content analysis tool assessed policies against a checklist of 26 items in three domains: (1) nutritional standards for a healthy food environment, (2) promotion of a healthy food and beverages environment, and (3) communication, implementation and evaluation of the policy. Domains were equally weighted, resulting in a score out of 30 [20]. Among those organisations included in the present study only eight had adopted the Policy, and so the remaining DHBs were grouped according to whether their organisational policy wording received a score above (strong, *n* = 5) or below (weak, *n* = 6) the average score of 18.6 out of 30 [20]. No organisation changed Policy adoption category during the study period.

### Characteristics and recruitment of participants

To be eligible, survey participants had to be a DHB or Ministry of Health staff or visitor (not in-patient), 18 years and older, able to provide verbal/written informed assent/consent and able to speak and understand English or te reo Māori (the Māori language).

To ensure the sample included participants from throughout New Zealand, we aimed to recruit at least 1000 staff and 1000 visitor survey participants across all organisations, with an initial target of 50 staff and 50 visitors per site. Recruitment was purposive, aiming to achieve at least 100 Māori and 50 Pacific staff, and 200 Māori and 100 Pacific visitors, in the total survey sample which may have led to more participants from regions with a higher proportion of Māori and Pacific peoples. Staff were invited to participate via an online link listed on the staff intranet or sent via email list, so that staff working shifts also had an opportunity to express their views and opinions. Two authors (SS and BK) promoted survey participation, both verbally and by leaving flyers and posters, whilst conducting audits of food/drink availability at cafes, staff cafetarias and vending machines within the organisations (data reported elsewhere, [22]). Staff and visitors were approached outside major food outlets and vending machines and asked if they would like to participate in the survey, either face-to-face and/or by a link to an on-line survey that could be completed remotely (provided on a study flyer). Due to Covid-19 protections in hospitals at the time, researchers were not able to recruit in-person as much as planned, and so when the researchers were not present onsite, and to accommodate people who preferred paper-based surveys, submission boxes with paper copies of the survey were placed in cafes and other venues, in collaboration with food retail staff. Additionally, to assist with meeting the target for Māori, Pacific or financially insecure visitors, a screening question about these characteristics was included. This screening question was only asked once the survey response target was achieved for the total number of staff or visitors at each site, and data was not collected from those who did not meet these criteria. However, due to promotion of the survey on staff intranets and via staff email lists, the staff targets for participation in many organisations were exceeded.

### Data collection process and materials

Electronic and paper-based surveys were undertaken from June 2021 to August 2022. All survey materials were available in English and Māori languages. Participants were informed on survey flyers, invitations, participant information sheets, and/or verbally that they were eligible to enter a draw to win one of five $100 shopping vouchers. To enter, an email address was requested at the end of the surveys but contact details were not associated with the responses from the survey.

The staff and visitor survey questions were based on surveys that measured the University of Auckland staff and student on-campus food/drink purchasing behaviours, and preferences and opinions on food/drink availability [23]. The following information was collected from all participants: (a) frequency of visiting an organisational food outlet, (b) satisfaction with the foods/drinks available for sale, (c) use of nearby shops or other food/drink sources within the organisation (e.g. vending machines and cake stalls), (d) awareness of, and attitudes to, the Policy, (e) any changes noticed since adoption of the Policy, (f) participant characteristics (age, gender, ethnicity, financial security, employment status, and occupation). Additionally, visitors were asked their reason for being in the healthcare facility and their plans for purchasing food on site. The survey was designed to be completed within 10 min.

All survey responses were collected and managed using REDCap software hosted at National Institute for Health Innovation, University of Auckland [24, 25]. Responses completed on paper-based forms were entered into REDCap manually by one author (BK).

### Data analysis

Categorical variables were presented as frequencies and percentages and compared between groups using the chi-squared test. Continuous variables were presented as mean and standard deviation, and compared between groups using the analysis of variance test. A *p*-value below 0.05 was used as evidence against the null hypothesis, suggesting a statistically significant difference. To provide an equity-lens in the analysis, the survey results were compared by level of adoption of the Policy (i.e., whether the organisation they were a staff or visitor at had adopted the National Healthy Food and Drink Policy, had their own comprehensively-worded organisational policy, or had a weaker organisational policy), and by ethnicity and financial security. Quantitative analysis was performed using SAS v.9.4 (SAS Institute Inc., Cary, NC, USA).

Free-text data was analysed using a qualitative general inductive approach–underpinned by the pragmatism research enquiry [26]–to assess staff and visitor awareness, support, and opinions about healthy food/drinks policies in New Zealand. All responses were read multiple times by one author (MR) to enable familiarisation with and immersion in the data. Free-text data were then coded inductively without an a priori framework in a Microsoft Excel spreadsheet containing the corresponding closed question responses for each participant. Each free-text record was allowed to fit into more than one descriptive or interpretative code. The codes were continuously revised as the analysis progressed and shared with another author (SM) to cross-check for consistency of interpretation. Responses that were deemed too general to deduct the intended meaning, or that referred only to the in-patient food service or hot drink provision (not covered by the Policy) were not included in the analysis. The codes and candidate topic summaries were discussed by two authors (MR and SG) for alignment with the study aims and relation to the quantitative results. Qualitative analysis results are presented in conjunction with the corresponding quantitative measures and as descriptive topic summaries.

## Results

### Survey respondent characteristics

The survey was completed by 2,526 staff and 261 visitors (2,549 online and 238 on the paper-based form, with four participants completing the questionnaire in te reo Māori), meeting the targeted recruitment numbers for staff overall, and targets for Māori and Pacific staff, but not visitors.

Table 1 describes the characteristics of participants. Most were female (82.9% of staff and 69.7% of visitors), non-Māori/non-Pacific ethnicity (85.1% of staff and 79.7% of visitors), and 28.0% of staff and 35.6% of visitors reported they were financially boarderline or insecure. There was a diversity of age-groups and regions represented in both the staff and visitor responses (Table 1).

**Table 1:** HYPE staff and visitor survey participant characteristics

Characteristic		Staff participants, *n* (%) *N* = 2526	Visitor participants, *n* (%) *N* = 261
Gender	Female	2095 (82.9)	182 (69.7)
	Male	389 (15.4)	71 (27.2)
	Gender diverse	14 (0.6)	3 (1.1)
	Missing / declined	28 (1.1)	5 (1.9)
Age group	18–24 years	178 (7.0)	21 (8.2)
	25–34 years	646 (25.6)	46 (17.9)
	35–44 years	521 (20.6)	38 (14.8)
	45–54 years	592 (23.4)	57 (22.2)
	55–64 years	495 (19.6)	44 (17.1)
	65 years or older	94 (3.7)	51 (19.8)
Ethnicity	Māori	318 (12.6)	47 (18.0)
	Pacific	58 (2.3)	6 (2.3)
	Other	2150 (85.1)	208 (79.7)
Financial position	Borderline or insecure	707 (28.0)	93 (35.6)
	Secure	1454 (57.6)	140 (53.6)
	Declined / missing	365 (14.4)	28 (10.7)
Work situation	Full-time	2023 (80.1)	97 (37.2)
	Part-time	456 (18.1)	30 (11.5)
	Self-employed	8 (0.3)	30 (11.5)
	Unemployed	1 (0.0)	22 (8.4)
	Retired	2 (0.1)	48 (18.4)
	Student	13 (0.5)	14 (5.4)
	Other / missing	23 (0.9)	20 (7.7)
Region	Northern North Island	692 (27.4)	23 (8.8)
	Te Manawa Taki (North Island)	432 (17.1)	64 (24.5)
	Central North Island	503 (19.9)	86 (33.0)
	Te Waipounamu (South Island)	823 (32.6)	78 (29.9)
	Central government (Ministry of Health)	76 (3.0)	10 (3.8)

Of the 19 organisations included in the study (18 DHBs and the Ministry of Health), eight had adopted the National Healthy Food and Drink Policy, five had a strongly-worded organisational policy, and six had a weakly-worded organisational policy (as described in an earlier publication [20]). Among staff participants, 42.5% (*n* = 1073) worked at an organisation with a weakly-worded policy, 21.5% (*n =* 543) worked at an organisation with a strongly-worded policy, and 36.0% (*n =* 910) worked at an organisation that had adopted the National Healthy Food and Drink Policy. Nearly half of visitor participants (*n =* 126, 48.3%) were visiting an organisation with a weakly-worded policy, 24.1% (*n =* 63) an organisation with a strongly-worded policy, and the remaining 27.6% (*n =* 72) were visiting an organisation that had adopted the National Healthy Food and Drink Policy.

Over 80% of staff (*n* = 2023) worked fulltime, employed as nurses or midwives (27.9%), administration staff (23.2%), allied health workers (21.9%), doctors (8.7%), management (6.8%), support staff (2.5%) or other (8.8%). 50% had worked at the organisation for five years or longer (*n* = 1255), 35% 1–4 years (*n* = 889) and 15% for less than a year (*n* = 365). One-third of staff were involved in shift work (*n* = 851, 33.9%).

Visitors to the organisations had a range of employment situations (Table 1), and 18.4% were retired. Visitors to the organisation had varied reasons for attending: 35.6% were having a procedure or clinic appointment, 28.4% were visiting a patient, 7.7% were attending a work meeting, and 25.7% were there for another reason (for example, being a student, contractor, or purchasing food).

### Current food and drinks purchasing behaviours

**Fig. 1:**
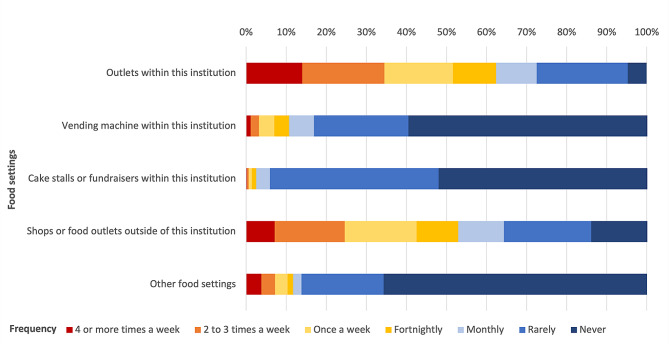
Frequency of staff participants (*n* = 2526) purchasing food and/or drinks from different settings within and outside of the organisation

Figure 1 presents the frequency with which staff buy food and drinks from various places inside and outside the organisations. Outlets within the premises were the most common place to purchase food (Fig. 1). Over half of staff purchased food or drinks at least once a week (14% four or more times a week) from outlets within the organisation (Fig. 1). Māori (*n* = 128, 40.6%) and Pacific (*n* = 30, 51.7%) staff were more likely to buy foods and drinks from on-site food outlets two or more times a week compared to other staff (*n* = 708, 33.2%, *p* = 0.0007). Purchases from external outlets while at work more than twice a week were also higher amongst Māori (*n* = 97, 31.0%) and Pacific (*n* = 20, 34.5%) staff compared to non-Māori/non-Pacific staff (*n* = 494, 23.4%, *p* = 0.0028). Seventeen sites had a total of 138 vending machines, but these were reportedly not used often for food and drink purchases, with over 80% of staff reporting that they never or rarely used them (Fig. 1). Explanations provided for infrequent usage of vending machines included the lack of healthy options or satisfactory food choices, low stock levels and absence of vending machines (at 3 of the 17 facilities).

Night shiftworkers were not well catered-for. Staff who worked shifts (*n* = 851) were asked where they purchased food during an evening or night shift. Over 80% (*n* = 701) brought their own food and drinks from home because there were limited options available during their shift hours and/or when their meal breaks were scheduled. However, 40.2% of shift workers (*n* = 342, about one-third of whom were doctors) consumed food from outlets within the organisation and some junior doctors and doctors reported that they received meals when on night-shift. The proportion of night shift workers using onsite vending machines ranged from less than 5% in half of the organisations to 40% in one region.

Nearly one in three staff who did shift work bought food from an outlet offsite (*n* = 249, 29.3%), and this was more likely among staff identifying as Māori (*n =* 43, 43.9%) or Pacific (*n =* 6, 46.2%) compared to staff of other ethnicities (*n =* 200, 27.0%, *p* = 0.0002). Reasons given for purchasing food offsite related to the opening hours: “*If you can’t get to the café on time you miss out and it is easier to go to a local alternative*” (Māori nurse/midwife). Pacific shiftworkers reflected on the limited availability of culturally appealing and affordable food choices, noting, for example, “*rice is available or potatoes*,* no taro to eat for Pacific Island people or Polynesian recipes. Seafood could be on the menu too*“ (support staff). Other sources of food during shiftwork included takeaways from delivery services, or food brought from home to share with co-workers. Onsite food options were particularly unsatisfactory for staff working nightshifts due to the high cost of snack foods, which were often the only foods available. Some shift workers indicated a need for “*that sugar hit*,* especially on a hectic night shift*” (allied health worker) and that “*It is important that comfort foods [are] also an option*,* particularly for tired and stressed staff who may need a pick me up*” (allied health worker). The need for sugary or comfort food comments were often associated in the free-text responses with a reflection on the highly stressful nature of shift work and burnout associated with working long hours.

Hospital food premises were the main source of foods and drinks for visitors. Three in every four visitors (*n* = 192, 75.0%) reported that they were planning to or had already purchased food and drinks during their current visit to the organisation. Nine out of ten of these visitors (*n* = 176, 91.7%) bought food and drinks from outlets within the organisation; 10.4% (*n* = 20) reported buying food and drinks from outlets outside the organisation, and 7.8% (*n* = 15) used vending machines. The factors which visitors reported affected their choice of food and drinks products (from a predefined list) were convenience (*n* = 106, 55.2%), tastiness (*n* = 71, 37.0%), price/value for money (*n* = 58, 37.2%), healthiness (*n* = 45, 23.4%), appearance of food/drinks (*n* = 48, 25.0%), comfort/feeling from foods/drinks (*n* = 44, 22.9%), and familiarity of foods/drinks (*n* = 34, 17.7%). There was positive feedback about the variety of options available in some DHBs, but it was frequently noted that these choices were expensive. As one visitor stated, “*The hot food available at the hospital cafe has improved quite a bit - more choice*,* better quality and is very good value. However I just can’t afford it at present*.” Visitors frequently noted difficulties in finding the sorts of foods they wanted to purchase, particularly foods for special dietary needs, culturally appealing foods, or foods sold in sustainable packaging. With regard to sustainability, visitors expressed concerns about single use coffee cups and plates, plastic food wrappings, and a lack of dedicated recycling bins.

### Awareness of healthy food and drink policy

A higher proportion of staff were aware that the organisation had a healthy food and drink policy (*n* = 1986, 79.3%) compared to visitors (*n* = 142, 56.3%). There were no statistically significant differences in awareness of the Policy among different ethnic groups (*p* = 0.28) nor based on financial situation (*p* = 0.58). Staff from organisations that had a strong healthy food and drink organisational policy (*n* = 453, 84.0%) were more likely to be aware of the policy compared with those that had a weaker organisational policy (*n* = 809, 76.1%, *p* = 0.0002). Respondents identified the need for positive communications about the Policy and promotion of healthier options to customers. As described by an allied health worker: “*[it’s] all about bringing people on a journey to get buy in*,* so there should be some more info around to inform them why there is no deep-fried food*,* big portions etc. Make it fun and light-hearted*”.

### Degree of support for a healthy food and drink policy

**Fig. 2:**
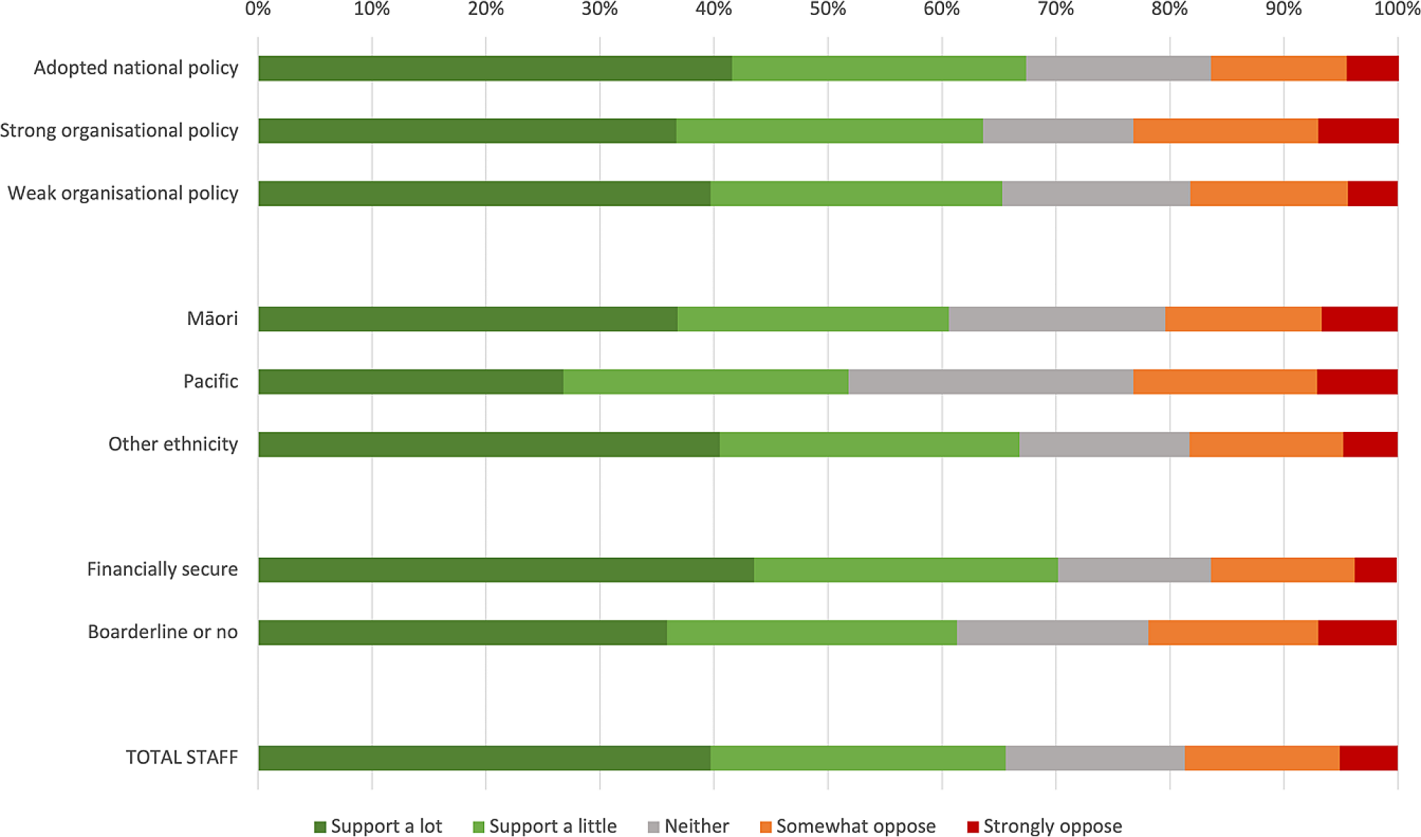
Staff support for healthy food and drinks guidelines at health organisations in New Zealand (*n* = 2526)

Both staff and visitors generally supported having organizational guidelines for healthy food and drinks (*n* = 1635, 65.6% of staff and *n* = 190, 76.3% of visitors), irrespective of whether they were aware of the policy or not. A greater proportion of staff in organisations that had adopted the National Healthy Food and Drink Policy supported having a policy, compared with those in organisations that had their own organisational policy (Fig. 2). Amongst those that opposed having organisational guidelines, a Pacific participant described “*Despite having healthy options*,* they can be somewhat overpriced which is not encouraging. It encourages me to walk outside for cheaper and unhealthier options*”. Generally, Pacific participants, and those who were borderline or financially insecure, were less likely to support a healthy food and drink policy compared to non-Pacific/non-Māori (*p* = 0.02) and those with greater financial security (*p* < 0.0001), respectively (Fig. 2).

### Reasons for supporting a healthy food and drink policy

Among staff who supported the healthy food and drinks guidelines (*n* = 1635), the most common reason selected was that “Hospitals should be good role models” (*n* = 1338, 81.8%). Approximately one third each of staff selected “Hospitals should not sell food/drinks that can lead to health problems” (*n* = 593, 36.3%) and “Hospitals should not sell food/drinks that are unhealthy” (*n* = 524, 32.0%) as reasons for supporting the guidelines. A small number (6.0%) gave ‘other’ free-text answers including: “*For the health of the staff*” (nurse/midwife) and that “*All public sector organisations should actively design healthy and sustainable food environments to ease the burden on whanau for accessing great and nourishing food*” (allied health worker). Some concerns were raised by those supporting the Policy. An administrator noted “*Such strict implementation … should also be … in conjunction with Local Council zoning that does not allow unhealthy food and drink retail outlets within a certain radius of hospitals to be effective and not just drive paying customers away from hospital outlets.*”

Fewer Māori and Pacific (*n* = 68, 24.5%) participants compared with non-Māori/non-Pacific (*n* = 525, 33.2%) agreed with the statement “Hospitals should not sell food/drinks that are unhealthy” (*p* = 0.01). Feedback received included comments about the importance of individuals having choices and the risk that high prices would drive people to go elsewhere to purchase cheaper, less healthy foods. As articulated by a Māori nurse, “*Hospitals need to be role models in promoting health food and drink*,* but a selection of not so healthy should be available or I’m just going to go next door and get whatever I want.*” Others commented on the unmet need for affordable, culturally appropriate food options onsite. For example, a Pacific administrator noted, “*Whilst I agree with the sentiment that we should be role modelling healthy eating*,* for the communities we serve the options are very limited and very expensive*”.

### Reasons for opposing a healthy food and drink policy

Among staff who opposed the healthy food and drinks guidelines (*n* = 465), the most common reason selected for doing so was “I should be free to eat what I want” (*n* = 313, 67.3%) and nearly half also selected “Foods that provide me with energy/comfort have been removed” (*n* = 221, 47.5%) as a reason for opposing the guidelines. Fewer participants agreed with the statement “The guidelines will be ineffective” (*n* = 71, 15.3%) as a reason. Free-text responses were provided by 70 (15.9%) participants. These included the need for more treat options to be available; insufficient alternative offerings at food outlets; high cost and low appeal of available foods: “*[the Policy has] resulted in bland food with the prices having increased significantly across the board*” (admin staff). Some respondents criticised the policy as “*environmentally irresponsible*” (doctor) and not “*evidence based*” (IT staff). Respondents also mentioned inconsistencies with removal of sugary drinks but not foods, and a lack of options for those with allergies or special dietary requirements.

### Changes in the food and drinks environment since adoption of the policy

**Fig. 3:**
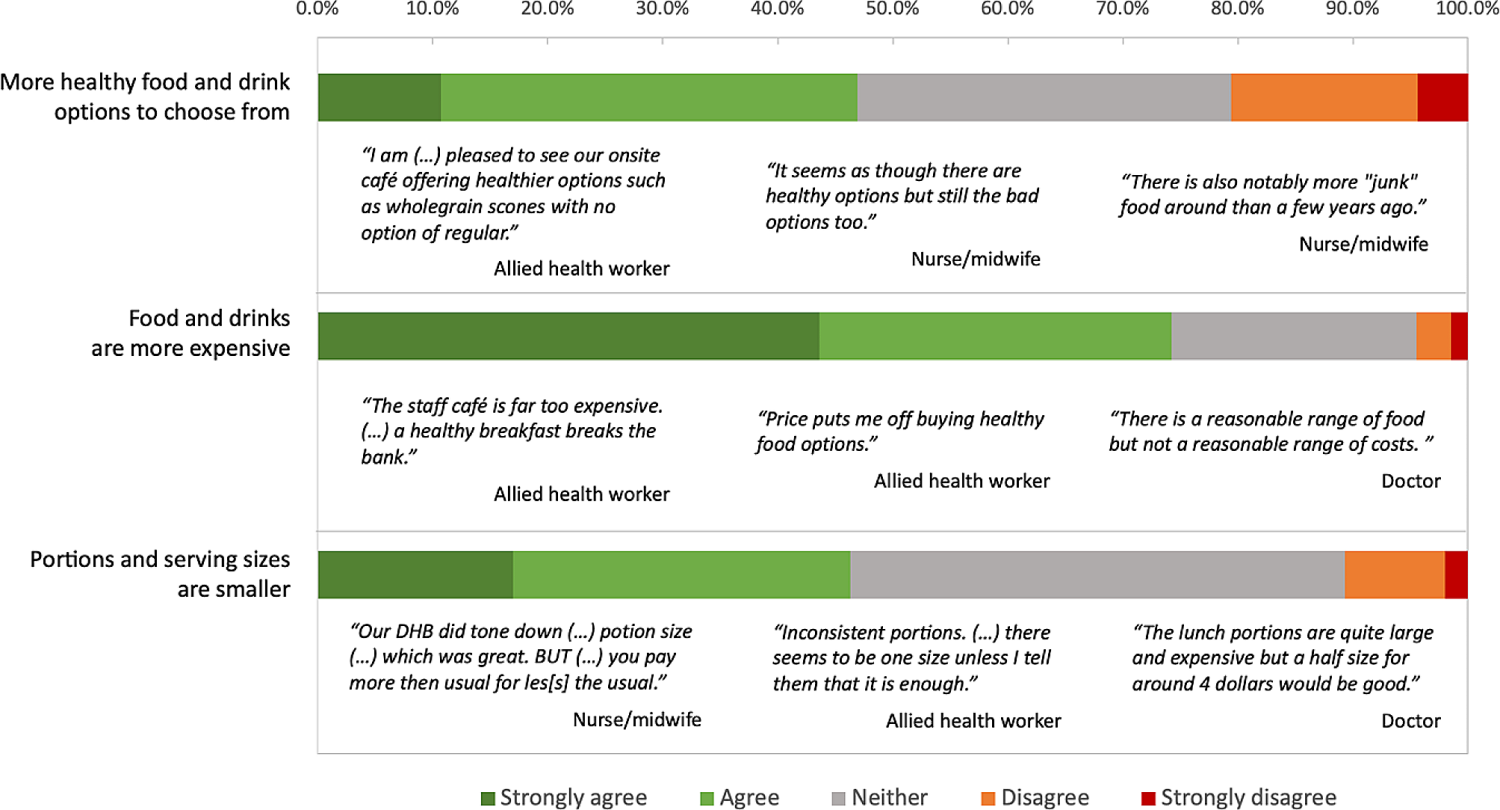
Changes in the food and drink environment noticed by staff (*n* = 2526) in the past year (following adoption of the Policy)

Staff were asked if they had noticed any changes in specific aspects of the food and drinks environment in their workplace over the past year (Fig. 3). Over 60% (*n* = 1532) agreed or strongly agreed that that there was less confectionery and/or sugary drinks available, 50% (*n* = 1274) agreed or strongly agreed that there was less deep-fried food, and nearly 50% agreed that there were more healthy food and drink options to choose from (*n* = 1175) and portion sizes were smaller (*n* = 1159). One participant had “*seen a whole vending machine with water inside (that previously would have had other drinks)*” (allied health worker). Some staff “*note[d] that these changes have been happening over much longer than the past year*” (allied health worker). Staff from organisations that had a strong healthy food and drink policy (*n* = 291, 53.8%) and those that had adopted the National Healthy Food and Drink Policy (*n* = 424, 47.0%) were more likely to agree or strongly agree that there were more healthy food and drink options to choose from, compared to those with weaker policies (*n* = 460, 43.2%, *p* = 0.0003).

However, 74.2% of all staff agreed or strongly agreed that food and drinks were more expensive, and only 4.9% agreed that food and drinks were now more affordable (Fig. 3). Staff also commented in the free-text responses on the reduction in variety of food available, higher prices, smaller portions, fewer portion size options and more plastic packaging as a result of the Policy. Staff that were financially borderline/insecure (*n* = 589, 83.5%) were more likely than those financially secure (*n* = 990, 68.6%, *p* < 0.0001) to report that foods and drinks had become more expensive, which resulted in people making less healthy choices or going to nearby outlets. As described by a financially insecure participant, “*hot meals for lunch over $10*,* salads have gotten smaller. The cheapest thing to buy is pies. So I buy pies*”.

### Staff and visitor satisfaction with food and drinks available

The mean satisfaction score for the food and drink available, on a scale of 1 (very dissatisfied) to 10 (very satisfied) was 4.7 (*SD* 2.3) for staff and 6.5 (*SD* 2.5) for visitors. Satisfaction with the foods and drinks available was lowest amongst Māori staff (4.5, *SD* 2.3) and those who were borderline or financially insecure (4.4, *SD* 2.3).

Two-thirds (*n* = 1774, 63.7%) of participants provided free text responses, and this feedback was predominantly negative. However, positive and negative comments were often contained within the same responses, especially among staff in organisations with more than one food provider. A third of respondents (*n* = 593, 33.4%) indicated that food options were either overly expensive (“*I’m not actually fussing with food but I end up taking mine from home as I hate paying top dollar for minimal product*” (nurse/midwife)), or not good value for money (“*A basic cheese sandwich now costs $8.50. That’s two slices of bread and one slice of cheese! How on earth can visitors to the hospital afford this?*“ (admin staff)). Only a small proportion of participants (*n* = 20, 1.1%) thought that some options were reasonably priced, and noted that these were mostly unhealthy options, hot dishes, and foods only available in the staff cafeteria (not for visitors). Overall, high prices were seen as a barrier to healthy eating.

The most common criticisms related to the lack of variety on offer (*n* = 714, 40.3%). While a small number of participants wanted red items to be reintroduced (e.g. sweetened drinks or chocolate), many more respondents wanted to see more fresh fruits, tasty vegetable sides, fresh non-carbohydrate-based salads, freshly made sandwiches and wraps, hearty soups in winter, more balanced protein/carbohydrate meals, and other healthy cold drink options in addition to water. Moreover, both staff and visitors pointed to limited or lack of vegetarian and vegan choices (*n* = 85, 4.8%), clearly labelled allergen-free options (especially gluten-free and dairy-free choices) (*n* = 22, 1.2%), and safe foods for pregnancy (*n* = 6, 0.4%). “*When there are [allergen-free] options*,* they tend to be poorly signed and staff members at the eateries do not know what is in the food - I find it safer just to avoid and go hungry!*” (allied health worker).

## Discussion

This study found staff and visitors throughout New Zealand’s healthcare organisations were aware of and broadly supported a healthy food and drink policy to improve their workplace/hospital food environments. The high participation rate of staff in this study suggests that the Policy is something hospital staff are keen to have input on. Australian studies of healthcare workers [27], hospital staff and visitors [28], food outlet managers and parents [7] and the general public [29] found similar strong support for making healthy meals more accessible and affordable onsite in healthcare services. An online survey of 20,000 adults in Australia, Canada, Mexico, the United Kingdom (UK) and the United States (US) found most support for food policies that provided incentives (e.g., price subsidies) or information (e.g., calorie labelling on menus), and least support for those that imposed restrictions on availability or choice [30]. A main complaint from staff about the Policy in the present study also related to the removal of unhealthy options without providing suitable alternatives, thus resulting in limited variety.

Around half of staff reported observing a greater availability of healthy foods and drinks, and less confectionary, deep-fried foods and sugary drinks since the Policy was introduced. However, some noted that unhealthy foods were still readily available and there was a lack of variety and options for those with dietary restrictions or allergies. On-site audits of all food and drink products available in the same organisations (as part of the independent HYPE evaluation) found that the Policy had failed in its aim to ensure that healthier food and drink options make up the majority of choices available in hospitals [22]. No organisation met the Policy criteria in terms of proportions of green, amber, and red items available to staff and/or visitors. Almost two in five (39.6%) of the food and drink choices available for staff and/or visitors were red items, which the Policy states are not permitted at all. In contrast, less than one in four (22.7%) were green items, which the Policy specifies should make up at least 55% of food and drinks available for consumption [22]. Organisations that adopted the National Policy had healthier foods and drinks on average compared to those with their own organisational policy, but the proportion of red items remained high: 33% versus 48% (*p* < 0.0001) [29]. The audits confirmed staff and visitor feedback received in the present study that there were few healthy options available onsite in some health care organisations in New Zealand, and inconsistency in implementation of the Policy.

The majority of staff in the present study reported that the price of food and drinks had increased since the Policy was introduced, and this echoes comments of workers in other studies about the high cost of food in hospitals [27]. As noted by many of the participants in our study, food price influences consumption [31] and high prices make it difficult for people to make healthier choices regardless of their initial intentions to select healthy food and drink. The higher food prices may have been due to inflation which was increasing over the study period. However, a review of 29 studies of hospital cafeterias found healthy menus *are* more costly than usual menus, but a healthy menu may increase sales if price discounts and reward-point systems were made available [21]. Abdul Rais et al. also noted that management may need to increase the promotion of healthy items on menus to increase customer demand and invest in food service staff training for better comprehension of the policy [21]. Increasing customer demand for healthy food was found to be critical to secure confidence that policy changes were sustainable in a systematic review of barriers and facilitators to the implementation of healthy food and drink policies [32]. The authors noted that by increasing customer demand, there was a decrease in “tensions between policy objectives and profitability” [32].

To be considered effective and equitable, a healthy food and drink policy should not only improve the food environment to shift the entire population (or average person’s) dietary behaviours, but ideally reduce the gap between the more and less advantaged groups, with greatest improvement for those who are most disadvantaged and at greatest risk of poor health outcomes [33]. Similar to Devine et al. [34], we found staff and visitors that were financially insecure were very concerned about limited food choice and increased costs due to Policy implementation, and this could limit the equity success of the Policy by excluding those on lower incomes who choose not to purchase food on site. This study also found that both the benefits and costs resulting from implementation of the Policy are likely to have impacted Māori and Pacific staff to a greater extent than non-Māori/Pacific staff because they reported buying food onsite more frequently than other staff. This suggests the Policy is pro-equity as it would help to reduce Māori and Pacific peoples greater risk of nutrition-related diseases. However, the Policy is undermined when foods and drinks provided are seen as being of poor value for money. Māori and Pacific staff noted that price increases meant they were more likely to choose the less healthy options that were cheaper, or to purchase food off-site where the Policy was not applied.

Another important consideration for equity is that staff and visitors to hospitals are often not in the best physical or mental health. Other studies have found nurses and allied health workers’ health to be poorer than the general population, with greater burden of chronic health conditions [35–37] many of which can be connected to extreme work-related strain [34]. Other researchers have noted that the high stress workload of hospital workers leads many to eat ‘emotionally’ and feel a need for ‘treat’ foods [38–40], a sentiment which was reflected in many of the free-text responses in the current study. Additionally, shift workers require special consideration when developing a healthy food and drink policy [10, 39] as it is particularly difficult to maintain healthy eating behaviours when circadian rhythms are interrupted [41] and food choices are limited outside traditional cafeteria operating hours as indicated by our participants. To achieve equitable outcomes, the Policy needs to specifically consider the needs of shift workers and those in suboptimal health states.

The findings from this study have clear implications for healthy food and drink policy implementation and public health practice which aims to promote healthy eating behaviours more broadly. A good communications plan, in place both during development and implementation of the policy, is vital to secure buy-in from staff and visitors [32]. Ongoing communications about the policy, for example, clearly visible posters about the changes and indicating healthier options on products, were requested. Importantly, the Policy needed to ensure a range of options for food and drinks were still provided (rather than simply removing unhealthy options). Special consideration should be made to make sure that a variety of healthier options were available for staff (including shift workers) and visitors, with diverse cultural and dietary restriction options. Most importantly, the healthiest options should be the most convenient, most appealing, and most affordable options available after the Policy is implemented. In order to ensure this, food providers and employers need to consider how discounts and promotions can be used to attract customers to the healthiest options and embed healthier behaviours.

The current study had several strengths and limitations that readers should note. Firstly, this survey was part of a comprehensive evaluation, including all district health board regions in New Zealand plus the Ministry of Health (with all DHBs participating in the Policy analysis, 19 DHBs with food and drinks audited and 18 DHBs where surveys of staff and visitors were conducted), with the data independently collected (not by DHB or Ministry staff). Additionally, a major strength was the large number and range of staff surveyed, whereas other similar research often only included one profession, e.g. nurses. The numbers of participants allowed for statistical differences between groups by ethnicity, financial security and strength of Policy wording to be included. However, the survey was conducted during the Covid-19 pandemic with multiple lockdowns and hospital entry restrictions, when staff were stretched and (as reported by food outlet staff) there were fewer visitors at hospitals than usual. This resulted in a small number of visitors surveyed despite multiple attempts to invite eligible people and, consequently, the statistics presented for visitors should be interpreted with caution. Gaps in the time period of when survey data was collected also occurred, due to to the Covid-19 public health effort preventing the study team from full engagement with each site. Another potential limitation of the study is that the majority of both staff and visitor participants were female. This may to some degree reflect the majority gender of both the healthcare workforce and hospital visitors but is also likely due to women being more likely to participate in research than men. It should also be noted that women are often considered to be, on average, more health conscious in high-income countries [28, 30] which could have led to a higher proportion reporting support for the Policy, than if the surveys had been representative of the entire New Zealand population.

## Conclusions

This national survey of hospital staff and visitors in 19 public healthcare organisations, found participants were largely aware and supportive of a healthy food and drink policy. Seven years on from the introduction of a National Healthy Food and Drink Policy, staff and visitors were somewhat satisfied with the available food and drink options and had noticed an improvement in healthy food/drink availability, but not affordability. The findings provide useful guidance on successful implementation of food and drink policies (e.g. providing more healthy food choices, engagement with staff, and keeping healthy options affordable for all customers) and suggests that the Policy could be expanded to other public workplaces. Using an equity lens in the analysis, we confirmed that the Policy had a greater potential positive impact on Māori and Pacific staff but was also likely to adversely impact them if (as found in our research) implementation did not prioritise culturally appropriate, affordable, healthy choices. Financially-insecure staff and visitors, and shift workers, were also particularly impacted by reduced choice and higher prices for healthy options. Survey respondents provided valuable suggestions for further improving the current food environments. Healthy food and drink policies in public sector workplaces have the potential to set a standard for nutritional quality in the food system – ensuring healthy food is the default option in all public settings – but they must be implemented with careful consideration of those most likely to be impacted by the changes.

## Data Availability

The datasets used and/or analysed during the current study are available from the corresponding author on reasonable request.

## Abbreviations

DHB: District Health Board
WHO: World Health Organization
